# Molecular signatures of host specificity linked to habitat specialization in *Exaiptasia* sea anemones

**DOI:** 10.1002/ece3.4058

**Published:** 2018-04-30

**Authors:** Emily S. Bellis, Reid. B. Edlund, Hazel K. Berrios, Harilaos A. Lessios, Dee R. Denver

**Affiliations:** ^1^ Department of Integrative Biology Oregon State University Corvallis Oregon; ^2^ Department of Biological Sciences Arkansas State University Jonesboro Arkansas; ^3^ Smithsonian Tropical Research Institute Balboa Panama

**Keywords:** 2bRAD, *Aiptasia*, Cnidaria, co‐evolution, population genomics, symbiosis

## Abstract

Rising ocean temperatures associated with global climate change induce breakdown of the symbiosis between coelenterates and photosynthetic microalgae of the genus *Symbiodinium*. Association with more thermotolerant partners could contribute to resilience, but the genetic mechanisms controlling specificity of hosts for particular *Symbiodinium* types are poorly known. Here, we characterize wild populations of a sea anemone laboratory model system for anthozoan symbiosis, from contrasting environments in Caribbean Panama. Patterns of anemone abundance and symbiont diversity were consistent with specialization of holobionts for particular habitats, with *Exaiptasia pallida*/*S. minutum* (ITS2 type B1) abundant on vertical substrate in thermally stable, shaded environments but *E. brasiliensis*/*Symbiodinium* sp. (ITS2 clade A) more common in shallow areas subject to high temperature and irradiance. Population genomic sequencing revealed a novel *E. pallida* population from the Bocas del Toro Archipelago that only harbors *S. minutum*. Loci most strongly associated with divergence of the Bocas‐specific population were enriched in genes with putative roles in cnidarian symbiosis, including activators of the complement pathway of the innate immune system, thrombospondin‐type‐1 repeat domain proteins, and coordinators of endocytic recycling. Our findings underscore the importance of unmasking cryptic diversity in natural populations and the role of long‐term evolutionary history in mediating interactions with *Symbiodinium*.

## 1 INTRODUCTION

Rising ocean temperatures threaten the persistence of coral reef ecosystems, invaluable hotspots of marine biodiversity that support the health and economy of human communities (Ferrario et al., 2014; Moberg & Folke, 1999). The fate of coral ecosystems is inextricably tied to the mutualistic symbiosis between corals and photosynthetic microalgae of the genus *Symbiodinium,* a hyperdiverse taxon classified into nine major clades designated A‐I (Pochon & Gates, 2010; Pochon, Montoya‐Burgos, Stadelmann, & Pawlowski, 2006). The coral‐*Symbiodinium* symbiosis funnels energy from sunlight through algal photosynthesis and into cnidarian metabolism to generate energy and the calcium carbonate framework of coral reef ecosystems (Gattuso, Allemand, & Frankignouelle, 1999). *Symbiodinium* living inside host endodermal cells receive access to carbon dioxide, inorganic nutrients, protection from herbivory, and a stable position in the water column to access light (Davy, Allemand, & Weis, 2012). In return, the coral host receives fixed carbon compounds that enhance calcification and support a large portion of host metabolic demands (Muscatine, Falkowski, Porter, & Dubinsky, 1984; Yellowlees, Rees, & Leggat, 2008).

Nutritional symbioses with *Symbiodinium* have facilitated the success of reef‐building corals in oligotrophic tropical waters, but may also render reef ecosystems particularly susceptible to global climate change (Kiers, Palmer, Ives, Bruno, & Bronstein, 2010; Six, 2009). Elevated temperatures just 1–2°C above summer maxima can trigger bleaching or breakdown of the symbiosis resulting in loss of *Symbiodinium* pigments or cells from coral host tissues through a variety of mechanisms (Weis, 2008). Widespread bleaching in natural coral populations is most commonly attributed to elevated temperature, through other stressors such as excessively cold temperature (Lirman et al., 2011), changes in salinity (Van Woesik, De Vantier, & Glazebrook, 1995), high solar irradiation (Gleason & Wellington, 1993), and nutrient enrichment (Vega Thurber et al., 2014) can induce or exacerbate bleaching. The frequency and severity of mass coral bleaching events are precipitously increasing (Heron, Maynard, van Hooidonk, & Eakin, 2016; Hughes et al., 2017).

A major obstacle in understanding these crucial symbiosis systems is the challenge of complementing ecological studies with basic insights from laboratory‐based approaches (Weis, Davy, Hoegh‐Guldberg, Rodriguez‐Lanetty, & Pringle, 2008). For many marine species, it is impossible to maintain large laboratory populations, propagate genetically controlled lines, or create different host–symbiont combinations. Because of these challenges, the sea anemone *Exaiptasia pallida* (Grajales & Rodríguez, 2014), commonly called “*Aiptasia,”* is emerging as a laboratory model system for studying coral symbiosis, particularly at the cellular level (Goldstein & King, 2016; Weis et al., 2008). These fast‐growing anemones reproduce sexually through broadcast‐spawning and asexually through pedal laceration, allowing large populations of genetically identical individuals to be propagated, experimentally bleached, and reinfected with cultured symbionts. Gene knockdown methods have been developed (Dunn, Phillips, Green, & Weis, 2007), and the lack of calcium carbonate skeleton facilitates in situ imaging impossible in adult reef‐building corals (Weis et al., 2008). Application of high‐throughput sequencing approaches to an experimentally tractable model system has offered a new window into the complex molecular dynamics underlying symbiosis onset and maintenance (Baumgarten et al., 2018; Matthews et al., 2017).

Despite the importance of *Exaiptasia* for molecular studies of cnidarian symbiosis, natural populations have rarely been studied. The first population genetic study, based on traditional markers, suggested two genetically differentiated populations: a “global” population that forms specific symbioses with *Symbiodinium minutum* (ITS2 type B1) and a South US Atlantic population that engages in diverse symbioses with *S. minutum, S. psygmophilum* (ITS2 type B2), and representatives of *Symbiodinium* clades A and C (Thornhill, Xiang, Pettay, Zhong, & Santos, 2013). A cryptic sister species, *E. brasiliensis,* has also been described from the southwestern Caribbean Sea and southwestern Atlantic Ocean but was only found in association with *Symbiodinium* clade A (Grajales, Rodríguez, & Thornhill, 2016).

At finer spatial scales in the Western Atlantic, patterns of symbiont association may be related to local variation in temperature and light environment. *Symbiodinium* clade A from *E. pallida* exhibited markedly increased photosynthetic performance at elevated temperatures compared to *S. minutum* (Goulet, Cook, & Goulet, 2005; Perez, Cook, & Brooks, 2001), a phenomenon that also depends on host genotype (Goulet et al., 2005). Correspondingly, anemone hosts experimentally infected (Perez et al., 2001) or naturally associated (Bellis & Denver, 2017) with *Symbiodinium* clade A tend to be less susceptible to heat‐induced bleaching. Higher rates of glucose transfer to hosts harboring *S. minutum* compared to *Symbiodinium* clade A, however, may indicate potential trade‐offs associated with increased thermal tolerance (see fig. 5a in Burriesci, Raab, & Pringle, 2012).

In this study, we investigate ecological and evolutionary dynamics of *Exaiptasia* anemones from four sites in Caribbean Panama where extensive long‐term environmental monitoring datasets are available. These locations are characterized by a variety of habitats and thermal regimes ranging from high daily variation often >3°C at a shoaling reef flat near the entrance to the Panama Canal (Cubit, Caffey, Thompson, & Windsor, 1989; http://biogeodb.stri.si.edu/physical_monitoring/research/galeta), to stable daily temperatures with means varying up to 1°C during the warmest months of the year among three mangrove lagoon sites in the Bocas del Toro Archipelago (Kaufmann & Thompson, 2005; http://biogeodb.stri.si.edu/physical_monitoring/research/bocas). To evaluate the hypothesis that environmental variation influences holobiont evolutionary dynamics at regional scales, we compare density and diversity of *Symbiodinium* from anemone populations at each of these sites. Potential ecological and evolutionary trade‐offs are assessed through estimation of anemone abundance and analyses of host genetic diversity based on type IIb restriction site‐associated DNA sequencing (2bRAD‐Seq; Wang, Meyer, McKay, & Matz, 2012). We further analyze 2bRAD‐Seq data to investigate locus‐specific patterns of host differentiation that could suggest potential roles in adaptation to local environments.

## 2 MATERIALS AND METHODS

### 2.1 Sample collection

**Figure 1:**
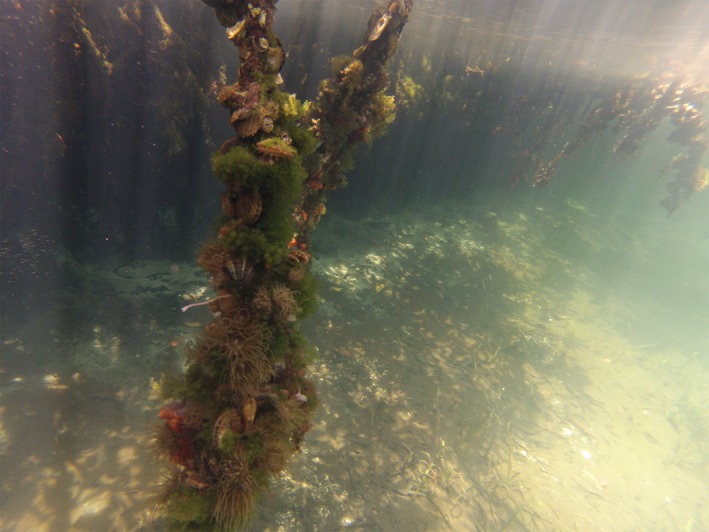
Mangrove root epibiota including *Exaiptasia pallida* sea anemones in Bocas del Toro, Panama

**Figure 2:**
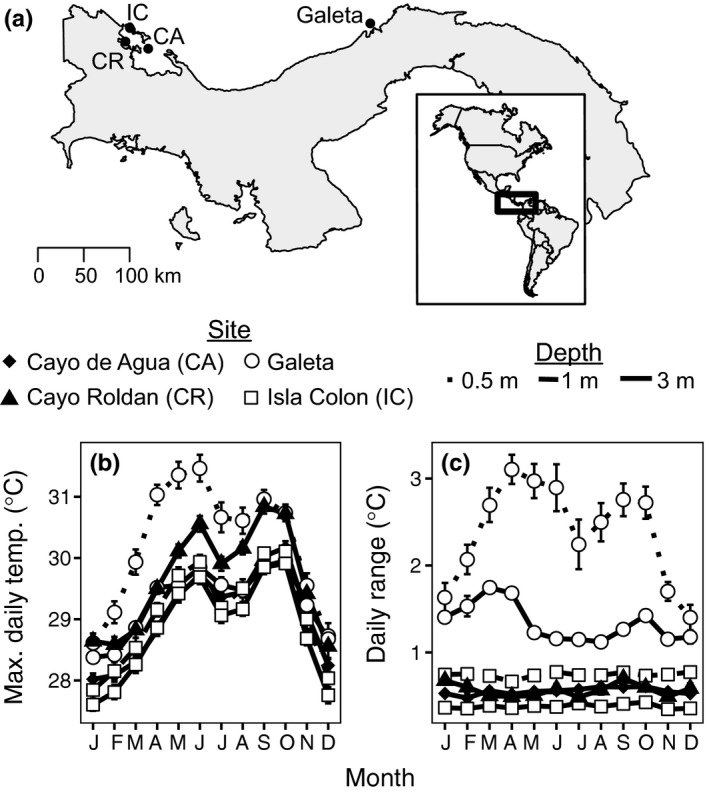
Primary sampling locations in Caribbean Panama. (a) Temperature data displayed in (b) and (c) are averages from 2000 to 2015 (3‐m depth datasets) or 2002 to 2015 (0.5‐m and 1‐m depth datasets) and are derived from data provided by the Physical Monitoring Program of the Smithsonian Tropical Research Institute

We surveyed populations of *Exaiptasia* sea anemones (Figure 1) across a range of habitats in Caribbean Panama. Surveys were conducted between 15 and 19 July 2015 (Galeta) or 30 August and 11 September 2015 (Bocas del Toro) at four sites: Isla Colón, Cayo Roldán, Cayo Agua, and Galeta (Figure 2). Survey areas were chosen based on proximity to long‐term water temperature monitoring sites operated by the Smithsonian Tropical Research Institute's Physical Monitoring Program. Several surveys were also conducted in Portobelo National Park, but as few anemones were found, this site was excluded for the majority of analyses. At each site, 30 m × 1 m belt transects were established parallel to shore. Transects were initiated upon observation of at least one anemone, and surveys were conducted continuously by an observer swimming along the shoreline. Abundance was estimated by counting all anemones per transect, with a maximum of 100 anemones counted per mangrove root. In Galeta, anemones were only found attached to rocks and coral rubble in shallow intertidal areas, so surveys were conducted parallel to shore at <0.5 m depth, with a maximum of 100 anemones counted per 30 m × 1 m belt transect. The median number of anemones per colonized root, the number of colonized roots in a transect, and the number of meters traveled before initiating the next transect were analyzed using generalized linear models (GLMs) and a quasi‐Poisson distribution to account for overdispersion with the “glm” function of R version 3.3.0.

### 2.2 Sample processing

Two anemones from each transect were sampled for genetic analysis and for determination of *Symbiodinium* density and mitotic index. Samples were homogenized in 200 uL buffer EB (Qiagen, Valencia, CA, USA), and a 100‐μl aliquot of the tissue homogenate was reserved for DNA extraction with the DNeasy Blood and Tissue Kit (Qiagen). The remainder of the homogenate was centrifuged at 5,000 g for 3 min, the pellet suspended in 500 uL filtered seawater (FSW), and the resuspension centrifuged again. The combined supernatants and the resulting algal pellet were frozen for determination of *Symbiodinium* cell density via hemocytometer counts and normalization to total protein determined using the Pierce BCA Protein Assay (see Appendix S1 for additional detail). Mitotic indices (MI) were estimated as the number of dividing cells per total number of counted cells for each replicate count. MI was analyzed using a generalized linear mixed model with the binomial distribution and logit link function implemented with the “glmer” function of the “lme4” package in R, which enables modeling of both fixed and random effects (Bates, Mächler, Bolker, & Walker, 2015). Site was included as the explanatory variable with a random effect of sample ID to take into account replicate counts (*n *= 6) from the same anemone. *Post hoc* comparisons were performed using the “glht” function of the “multcomp” package (Hothorn, Bretz, & Westfall, 2009).

### 2.3 Anemone genotyping

We created reduced representation sequencing libraries using DNA from 150 anemones from Caribbean Panama. These included anemones from Isla Colón (*n *= 42), Cayo Roldán (*n *= 38), Cayo Agua (*n *= 33), Galeta (*n *= 32), and Portobelo National Park (*n *= 5). To facilitate comparison with laboratory strains, we created additional libraries for four previously studied strains CC7, H2, EM5, and MMB (Bellis & Denver, 2017) and a single anemone, N2, from the outdoor aquarium at Naos Island Marine Laboratory in the Bay of Panama. In silico digested reads from low‐coverage whole genome sequencing of 10 laboratory *E. pallida* strains (Bellis, Howe, & Denver, 2016) were also included.

Genomic libraries were prepared and analyzed following the 2b‐RAD method (Wang et al., 2012). Genomic DNA was digested with the enzyme *BcgI* (New England BioLabs, Ipswich, MA, USA), which cleaves on either side of the recognition sequence ([N10]CGA[N6]TGC[N12]), to generate 36‐bp fragments. Dual‐indexed libraries were gel‐purified after adaptor ligation and amplification, pooled, and sequenced on the NextSeq 500 at the University of Oregon Genomics and Cell Characterization Core Facility (Eugene, OR, USA). After sequencing, reads were quality‐filtered and aligned to the set of all 44,428 *BcgI* recognition sites present in the v1.0 reference genome (Baumgarten et al., 2015) using SHRiMP v2.2.2 (Rumble et al., 2009). Genotypes with minor allele frequencies (MAF) below 5% were called as homozygous; those with frequencies above 30% were called as heterozygous. Genotypes were not called if MAF was between 5 and 30% or if coverage was <5×. Variants were further filtered to exclude sites not genotyped in at least 80% of individuals (see Appendix S1 for full methodological details).

Because *E. brasiliensis* and *E. pallida* are morphologically indistinguishable (Grajales & Rodríguez, 2016), species assignments were based on phylogenetic analysis (Figures S1‐S2). Phylogenetic trees were inferred using the maximum‐likelihood method based on the Kimura 2‐parameter model in MEGA7, with the discrete Gamma distribution (five categories, estimated α = 0.43) used to model evolutionary rate differences among sites and allowing for invariable sites. Individuals identified as *E. brasiliensis* were confirmed by sequencing a region of mitochondrial DNA that spanned the 16S ribosomal RNA (*16S rRNA*), cytochrome c oxidase subunit III (*COIII),* and cytochrome c oxidase subunit I (*COI*) genes from several individuals. This region exhibited an interspecific divergence of 0.32% over 3,164 bp, no intraspecific polymorphisms in a trimmed 719‐bp region sequenced in eight individuals, and included a region previously found to distinguish *E. brasiliensis* and *E. pallida* (Grajales & Rodríguez, 2016). Mitochondrial DNA was amplified using oligonucleotides MslrRNA‐F (5′‐CTGGAAACTGAAACATCTAAGTACC‐3′) and ApCOI_5‐R (5′‐TCATTCCAGAACCTATTCCAAA‐3′) each at a final concentration of 0.4 μmol/l in 50‐μl reaction volumes containing 1 μl DMSO, 10 μl 5 ×  MyTaq Reaction Buffer (5 mmol/l dNTPs, 15 mmol/l MgCl_2_), and 0.25 μl MyTaq DNA Polymerase (Bioline, London, UK). A PCR cycling protocol optimized for amplification of long DNA products was used as follows: 35 cycles of 92°C for 10 s, 55°C for 20 s, and extension at 60°C for 10 m with an additional 10 s per cycle added to the extension time after the first 10 cycles. PCR products were sequenced with BigDye Terminators on the ABI 3730 capillary sequencer at the Center for Genome Research and Biocomputing at Oregon State University (Corvallis, OR, USA) after cycle synthesis with oligonucleotides MslrRNA‐F, ApCOI_5‐R, and internal sequencing primers ApCOIII‐R (5′‐AGACCTTGGAAAGTTGCCTCT‐3′) and AplRNA‐R (5′‐AAGCTCACCTTCGTTACCTTT‐3′).

### 2.4 Anemone population structure

To investigate population subdivision, we performed hierarchical population structure analyses in STRUCTURE version 2.3.4 (Falush, Stephens, & Pritchard, 2003; Pritchard, Stephens, & Donnelly, 2000). To identify clones, we used “poppr” version 2.3.0, with genotypes contracted based on Prevosti's absolute genetic distance and clusters merged according to the maximum distance in each cluster (Kamvar, Tabima, & Grünwald, 2014). The threshold for clustering clonal genotypes (0.006) was chosen based on genetic distances observed for known clones in the dataset (HI1/HI2 and BM1/BM2 in silico digested libraries from Bellis et al. (2016); CC7 in silico and in vitro digested libraries). STRUCTURE analyses on the clone‐corrected dataset used the admixture model with correlated allele frequencies, which provides greater power to distinguish closely related populations compared with the independent allele frequency model (Falush et al., 2003). Five replicates were run for values of *K* ranging between 1 and 6, each with 500,000 MCMC steps after a burn‐in period of 100,000. The optimum number of population clusters, *K*, was determined using the Evanno method implemented in STRUCTURE HARVESTER (Evanno, Regnaut, & Goudet, 2005; Earl & vonHoldt, 2012; Figures S3–S5). Output from replicate STRUCTURE runs was combined with CLUMPP version 1.1.2 (Jakobsson & Rosenberg, 2007) and visualized using DISTRUCT version 1.1 (Rosenberg, 2004). To complement analyses of STRUCTURE, principal components were visualized with “adegenet” version 2.0.1, with allele frequencies scaled and centered to the mean and missing genotype calls replaced by the mean allele frequency (Jombart, 2008).

### 2.5 Locus‐specific patterns of genetic differentiation

To identify outlier loci in *E. pallida* most strongly correlated with divergence between local populations, we used the R package “pcadapt” version 3.0.4 (Luu, Bazin, & Blum, 2017). Component‐wise *p*‐values were computed retaining the first two principal components, chosen based on visual inspection of the scree plot, and false discovery rate correction with a threshold of 0.05. Gene set enrichment analyses were performed with the gene score resampling method on component‐wise *p*‐values describing the strength of SNP associations with PC1 in ErmineJ version 3.0.3 (Gillis, Mistry, & Pavlidis, 2010). Analyses were based on the median score for each gene set, with full resampling and 200,000 iterations, incorporating testing for multifunctionality. Weir and Cockerham's *F*_ST_ was calculated using VCFtools for each biallelic locus after removing alleles with frequency below 5% (Danecek et al., 2011).

### 2.6 Symbiont genotyping

The dominant *Symbiodinium* in each sample was genotyped as *S*. sp. clade A, *S. minutum* type B1, or *S. psygmophilum* type B2 using a two‐stage PCR banding assay. The first PCR reaction was specific for the chloroplast 23S ribosomal RNA (*cp23s*) from *Symbiodinium* clade B. Primers 5′‐TGC TGC TGA CAC TAA AGG ACA‐3′ and 5′‐ATC GCC CCA ATT AAA CAG TG‐3′ were designed to flank an insertion polymorphism between voucher specimens of *Symbiodinium* type B1 [GenBank accession: JN557991] and type B2 [GenBank accession: JN557995], yielding a 165‐bp band for type B1 or a 205‐bp band for type B2. The second PCR reaction was specific for the chloroplast photosystem II subunit D1 (*psbA*) from *Symbiodinium* clade A and did not amplify DNA from types B1 or B2 (primers 5′‐CTT TCT CAG CTC CAG TAG TT‐3′ and 5′‐AGA TTG AGT GAA ATG TCT CCT G‐3′). All PCRs were performed with EconoTaq DNA Polymerase (Lucigen, Middleton, WI, USA) in 25‐μl reaction volumes with an annealing temperature of 56°C.

## 3 RESULTS

### 3.1 Anemone distribution and abundance

We investigated the distribution and abundance of *E. pallida* and *E. brasiliensis* at four sites in Caribbean Panama (Figure 2). *E. pallida* was found only on vertical substrate and was most abundant on the roots of island‐fringing mangroves (~0.5–2 m depth) at the three studied sites within the Bocas del Toro Archipelago (Table 1). In contrast, *E. brasiliensis* was abundant in Galeta attached to rocks or coral rubble on a shoaling reef flat (≤0.5 m depth), although four *E. brasiliensis* specimens were found colonizing mangrove roots in the Bocas del Toro Archipelago (Table 1). No anemones were found attached to mangrove roots in Galeta or in Portobelo National Park.

**Table 1:** Summary of samples genotyped. Anemones are classified according to host species and the dominant *Symbiodinium* type

Site	Host species	Substrate	Number of anemones		
			*Symbiodinium* sp. (clade A)	*S. minutum* (type B1)	*S. psygmophilum* (type B2)
Bocas del Toro					
Cayo Agua	*E. pallida*	Mangrove	0	33	0
Cayo Roldán	*E. brasiliensis*	Mangrove	0	1	0
	*E. pallida*	Mangrove	0	37	0
Isla Colón	*E. brasiliensis*	Mangrove	0	3	0
	*E. pallida*	Mangrove	0	38	1
Coastal populations					
Galeta	*E. brasiliensis*	Reef flat	28	0	0
	*E. pallida*	Dock piling	0	4	0
Portobelo	*E. brasiliensis*	Reef flat	5	0	0

**Figure 3:**
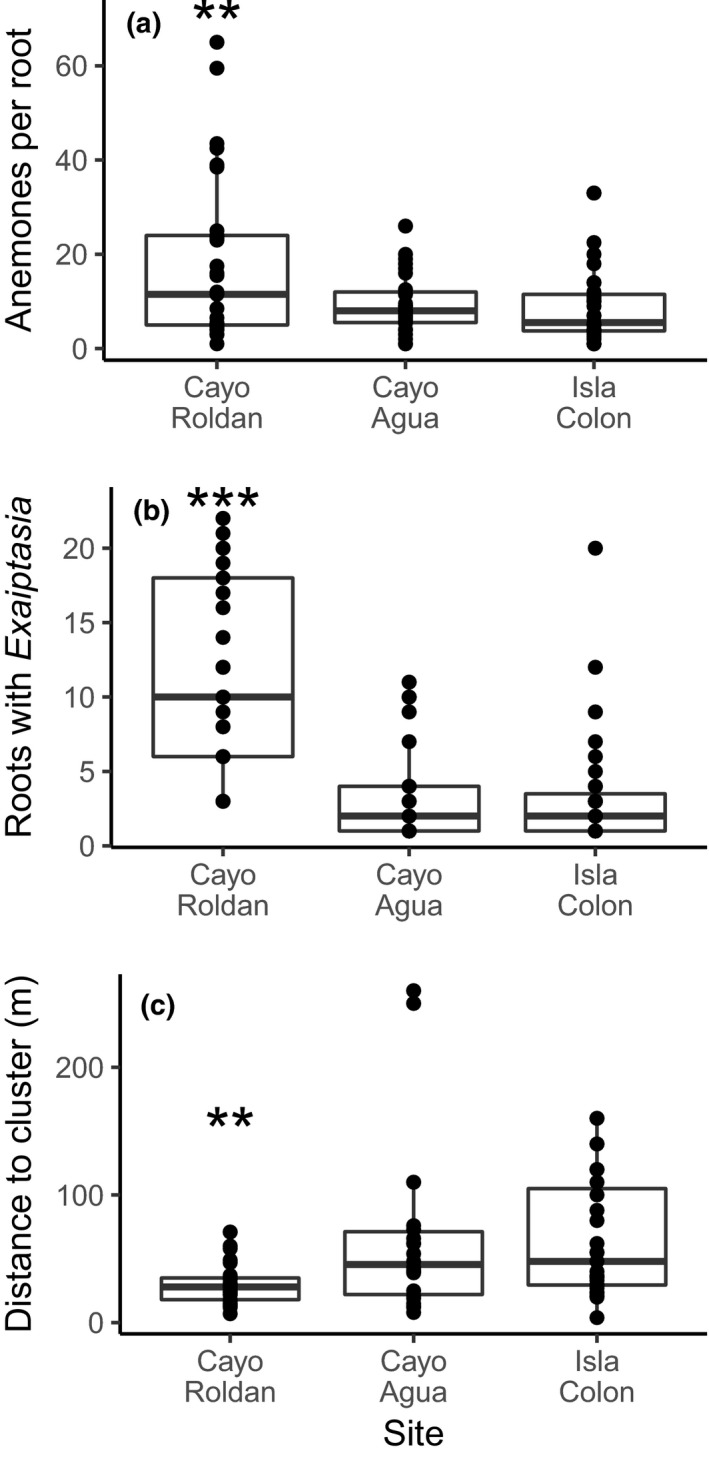
Anemone abundance in Bocas del Toro. Boxplots delimit the 25th and 75th percentiles, and the horizontal bar indicates the median. Data shown in a–c are based on continuous surveys, with 30‐m transects initiated upon observation of at least one anemone. (a) Median number of anemones per colonized mangrove root in each 30‐m transect. (b) Number of colonized roots per transect. (c) Distance between transects. Galeta is not shown, as no anemones were found colonizing mangrove roots, and the majority of individuals genotyped were *E. brasiliensis* rather than *E. pallida*. Asterisks indicate significant differences between Cayo Roldán and other sites (*p *<* *.001 [***] or *p *<* *.01 [**], generalized linear model)

Within the Bocas del Toro Archipelago, anemones were most abundant at the warmest site, Cayo Roldán (Table 2). A median of 18 anemones was observed per colonized mangrove root in Cayo Roldán, a higher density compared to other sites (*p *< .01, GLM; Figure 3a). In addition, a greater number of mangrove roots were colonized per transect (*p < *.001, GLM; Figure 3b), and transects, with placement dependent on anemone distance, were initiated more closely together at Cayo Roldán than the other sites (*p < *.01, GLM; Figure 3c). The distance between initiated transects in Galeta was similar to Isla Colón and Cayo Agua (data not shown).

**Table 2:** Statistical analyses of anemone abundance in Bocas del Toro. Count data for all response variables were modeled using generalized linear models assuming a quasi‐Poisson distribution to account for overdispersion

	Estimate	Std. Error	*t*‐Value	*p‐*Value
*Anemones per root*				
Intercept	2.89	0.14	20.85	<.001
Site				
Cayo Agua	−0.65	0.24	−2.68	.009
Isla Colón	−0.68	0.23	−2.91	.005
*Roots with ≥1 anemone*				
Intercept	2.47	0.10	23.76	<.001
Site				
Cayo Agua	−1.24	0.23	−5.51	<.001
Isla Colón	−1.21	0.21	−5.73	<.001
*Distance between clusters (meters)*				
Intercept	3.40	0.22	15.534	<.001
Site				
Cayo Agua	0.78	0.27	2.90	.005
Isla Colón	0.80	0.26	3.05	.003

### 3.2 *Symbiodinium* density and diversity

We analyzed the occurrence of three *Symbiodinium* species that associate with *E. pallida* (Grajales et al., 2016) using a PCR banding approach that targeted *cp23s* and *psbA*, two chloroplast genes encoded by separate minicircles (Wisecaver & Hackett, 2011). All sampled *E. pallida* hosted *S. minutum* (ITS2 type B1) as the dominant symbiont, with the exception of a single individual from the global *E. pallida* clade collected in Isla Colón that harbored *S. psygmophilum* (Table 1) *. E. brasiliensis* was dominated by *S. minutum* at sites within the Bocas del Toro Archipelago but *Symbiodinium* sp. clade A in Galeta (Table 1). Because *Exaiptasia* was previously reported to associate with multiple genotypes of *Symbiodinium* clade A (Grajales et al., 2016), we sequenced the *psbA* region for a subset of samples (*n *= 6). Four sequences were identical to a *Symbiodinium* clade A isolated from the jellyfish *Cassiopeia xamachana* [GenBank: AJ884905], whereas two sequences were identical to sequence for *Symbiodinium* isolated from a representative of the laboratory *E. pallida* strain used to construct the reference genome (Baumgarten et al., 2015). Although our PCR analysis does not preclude the presence of additional genotypes or finer scale genetic differences, in all samples, a single dominant *Symbiodinium* type was discovered, and PCR with alternative primer pairs failed to amplify a discernable band.

**Figure 4:**
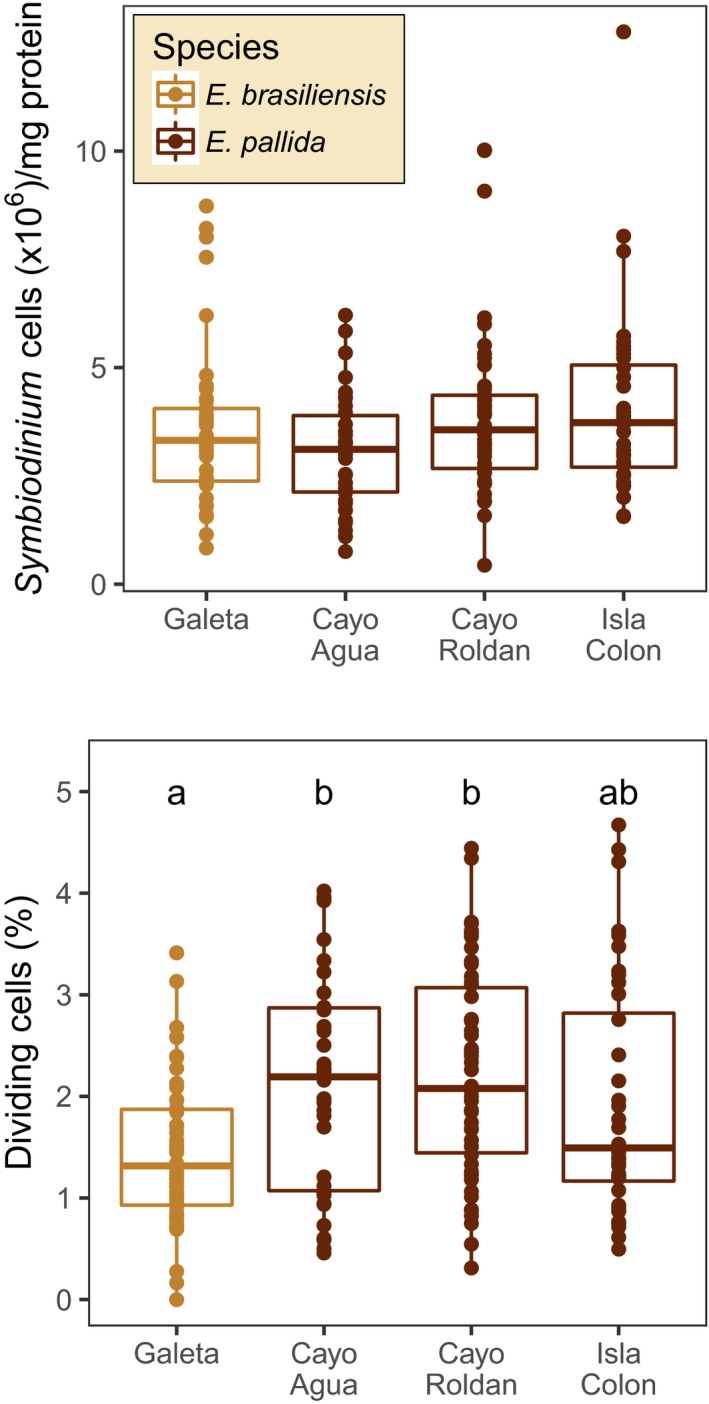
Variation in *Symbiodinium* cell density (top panel) and mitotic index (lower panel) among sites. Boxplots delimit the 25th and 75th percentiles, and the horizontal bar indicates the median. Strong evidence for differences in *Symbiodinium* density among sites was not observed (*p = *.08, generalized linear model). In contrast, site was a significant predictor of mitotic index (*p < *.001, GLMM), with evidence for lower *Symbiodinium* division rates in anemones from Galeta compared to Cayo Roldán (*p *<* *.001, Tukey HSD) and Cayo Agua (*p *=* *.014, Tukey HSD). Anemones from Galeta were primarily *E. brasiliensis* associated with *Symbiodinium* sp. clade A, whereas the majority of anemones from the other sites were *E. pallida* associated with *S. minutum* type B1. Because not all samples used for density analyses were genotyped, datapoints are colored according to the dominant anemone species at each site

Within‐anemone *Symbiodinium* cell densities per unit protein were similar across sites, but lower division rates were observed in *Symbiodinium* clade A in anemones from Galeta compared to *Symbiodinium* type B1 at other sites (Figure 4). Median density ranged between 3.1 and 3.7 × 10^6^ cells per mg protein across the four sites, with a median of 3.3 × 10^6^ cells per mg protein in the pooled sample set. There were no significant differences in within‐anemone *Symbiodinium* cell density among sites (*p = *.08, likelihood ratio test for full and intercept‐only model). However, site was a significant predictor of mitotic index (*p *<* *.001, likelihood ratio test for comparison of full and intercept‐only model). Average mitotic index of *Symbiodinium* clade A was lower in anemones from Galeta compared to *Symbiodinium* type B1 in Cayo Roldán (*p *<* *.001, Tukey HSD) or Cayo Agua (*p *=* *.014, Tukey HSD) (Figure 4).

### 3.3 Host population structure

We investigated population genetic structure of anemones from the Caribbean coast of Panama using type IIb restriction site‐associated DNA sequencing (2bRAD‐Seq). To facilitate comparison with previous studies, sequences from 15 *E. pallida* isolates collected from geographically widespread locations were also included. On average, 1.2 million reads were generated from each sample. An average of 51.5% (*E. brasiliensis*) or 64.2% (*E. pallida)* of reads mapped to the reference genome; significantly fewer reads from *E. brasiliensis* mapped uniquely compared to *E. pallida* (32.8% vs. 43.2%, *p *<* *.001, Welch two sample t test). Mapping was most successful for the 2bRAD library created from CC7, the strain used to sequence the reference genome (82%), but only 59% of CC7 reads mapped uniquely, suggesting that microbial DNA in the sequencing libraries, divergence from the reference genome, and the short length of the 2bRAD tags all contributed to lower mapping percentages. After filtering, 4640 SNPs were identified in the *E. pallida* callset and 5391 SNPs in the combined species dataset; of these, 2577 remained for *E. pallida* after removing sites with an allele frequency below 5%.

**Figure 5:**
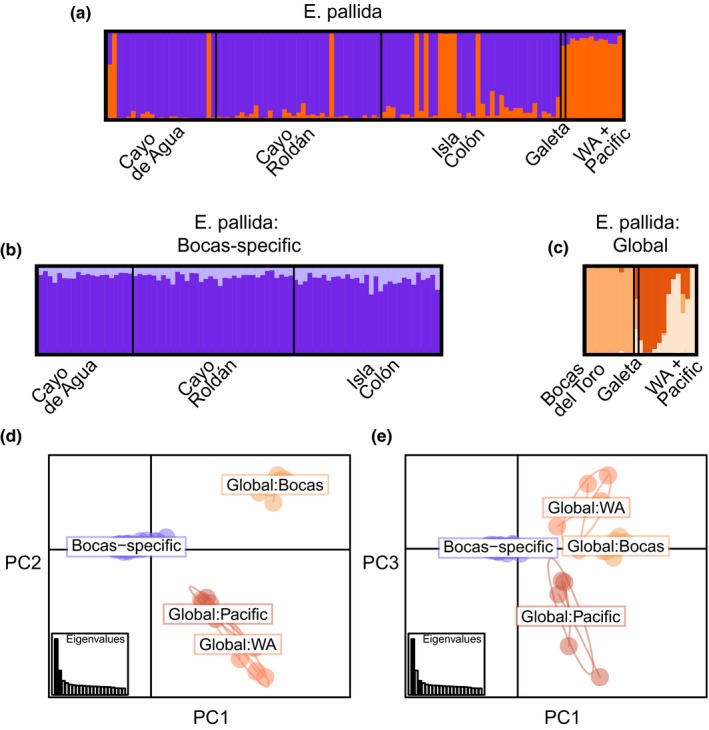
Structure of *E. pallida* Bocas‐specific and globally widespread populations. STRUCTURE analyses were first performed for all *E. pallida* samples (a). The two identified populations in (a) were then analyzed individually (b–c). The optimal number of clusters (*K* = 2 in a and b; *K* = 3 in c) was chosen based on the Evanno method (Evanno et al., 2005). Representatives of the previously described South U.S. Atlantic and global clades (Grawunder et al., 2015; Thornhill et al., 2013) are included in the Western Atlantic (WA) + Pacific grouping. Results of the principal component analysis (d–e) are shown after excluding three individuals with significant proportions of admixture from multiple populations. Analyses were based on 2,577 SNPs after removal of clones and anemones determined as *E. brasiliensis*

Patterns of genetic variation at 2,577 SNPs supported the presence of two distinct *E. pallida* populations: a local population specific to the Bocas del Toro Archipelago (which we name the “Bocas‐specific” population) and a separate population including individuals we collected in Panama that clustered more closely with geographically widespread *E. pallida* (which we call the “global population”) (Figure 5). The “global population” cluster in our study included anemones from Florida, Bermuda, and Hawaii as well as four clones from a dock piling in Galeta, ten individuals from sites in the Bocas del Toro Archipelago, and the anemone that was collected from the outdoor aquarium at Naos Island Marine Laboratory in the Bay of Panama (red vertical bars in Figure 5a). Our definition of a global clade, therefore, differs from that of a previous study (Thornhill et al., 2013), which included individuals from Bermuda, Hawaii, Japan, the Red Sea, and Australia but not Florida as part of the global *E. pallida* group.

**Figure 6:**
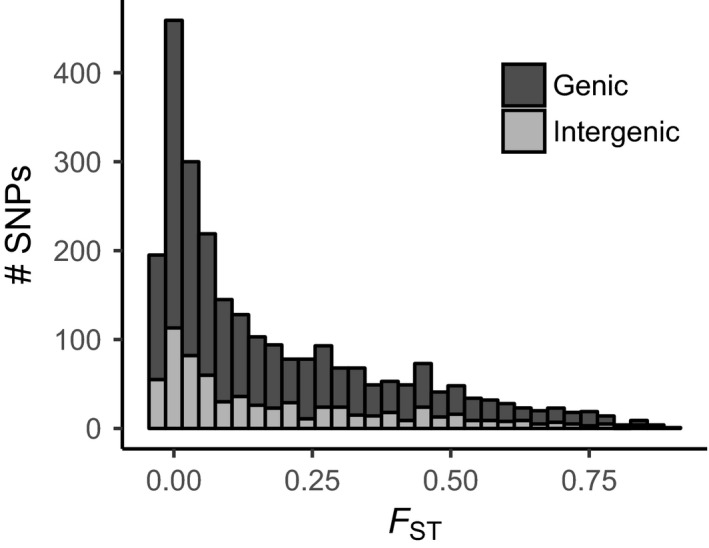
Distribution of pairwise *F*_ST_ values for comparisons between the Bocas‐specific and global *E. pallida* populations. Single nucleotide polymorphisms in genic regions (*n *= 1,881) include those present in introns, exons, and 5′‐ and 3′‐untranslated regions

A median *F*_ST_ value of 0.10 between the Bocas‐specific and the global clades was in agreement with the distinction of the Bocas‐specific and globally widespread *E. pallida* populations. The distribution of *F*_ST_ values among loci was positively skewed, with a similar proportion of SNPs in genic regions compared to the proportion of the reference genome covered by gene models (73% vs. 76%, respectively; Figure 6). Within the Bocas‐specific population, strong genetic differentiation among the three sampled sites in the Bocas del Toro Archipelago was not observed (Figure 5b). In contrast, subpopulation structure was indicated in the global population (Figure 5c), with a median *F*_ST_ of 0.06 between representatives of the global population collected in Bocas del Toro and individuals from globally widespread locations.

Because *Exaiptasia* can reproduce clonally through pedal laceration, we removed individuals genotypically identical to other samples in the dataset prior to analyses of population structure. The spatial extent of clonal reproduction in the populations studied appeared to be relatively limited. Among the 112 sampled *E. pallida* individuals*,* 87 multilocus genotypes (MLGs) were unique, seven MLGs were shared by two anemones, one MLG was shared by three anemones, and two MLGs were shared by four anemones. All anemone pairs genotyped from the same mangrove root or dock piling were clones (*n *= 6 pairs). Shared MLGs for *E. pallida* collected from different transects were observed between the members of two clone pairs from Cayo Roldán, a maximum distance of 50 m apart. Among individuals genotyped from different mangrove roots within the same 30‐m transect, 0%–29% of genotyped pairs were clones, including 0 of 11 transects in Isla Colón, one of 13 transects in Cayo Roldán, and two of seven transects in Cayo Agua. In *E. brasiliensis*, six clone pairs were identified and a total of 29 unique MLGs among 35 genotyped individuals. Members of four clone pairs were collected <30 m apart, whereas two pairs of clones were collected with their members approximately 100 m apart.

### 3.4 Locus‐specific patterns of host differentiation

Under a model of divergence among three populations, in which two populations share more recent evolutionary history compared to a third*,* outlier loci strongly correlated with principal components that separate these populations may be promising targets of differential selection pressure (Duforet‐Frebourg, Luu, Laval, Bazin, & Blum, 2016). To identify loci in *E. pallida* most strongly correlated with divergence between the Bocas‐specific and global populations, we performed outlier detection using principal component analysis (Luu et al., 2017). This analysis was based on 2577 SNPs that remained after removing biallelic sites with minor allele frequency below 5%. To further reduce false positives, we report only those loci that were also significantly associated with divergence between the Bocas‐specific and global *E. pallida* populations in comparisons using the Mahalanobis distance, which may provide higher power for hierarchically structured populations compared to other methods when there is admixture (Luu et al., 2017).

Five loci showed strong signatures of association with the first principal component (PC1) at an FDR threshold of 0.05 (Table 3). These included two sites in intergenic regions and three loci located in genic regions. One locus was identified in the coding region of cytoplasmic FMR1‐interacting protein 2, but was not predicted to cause a change to the protein sequence (Table 3). The nearest predicted gene to one of the identified intergenic sites was an uncharacterized protein approximately 1 kb away from this locus. The other intergenic site was in the midst of a ~4.7 kb intergenic region between genes identified as Frizzled‐5 and General transcription factor IIF subunit 2. While none of the identified polymorphisms were expected to result in changes to protein sequence, these sites could be linked to nonsynonymous polymorphisms, influence gene expression, or impact protein structure through alternative splicing (Table 3).

**Table 3:** Individual outlier loci associated with divergence of the Bocas‐specific population in the principal component analysis. *F*_ST_ values for each locus are also given and reflect comparisons between the global *E. pallida* population (including individuals from Bocas del Toro, Bermuda, Florida, and Hawaii) and the Bocas‐specific population

Scaffold	Position	Region	Identifier	*F*_ST_	Annotation
10	1258482	5′UTR	AIPGENE19408	0.85	Myoferlin
143	149323	Intergenic	N/A	0.90	N/A
227	50641	Intron	AIPGENE5586	0.83	*N*‐glycanase 1
47	283015	Intergenic	N/A	0.83	N/A
60	564146	CDS	AIPGENE172	0.86	Cytoplasmic FMR1‐interacting protein 2

Because individual outlier loci were present in genes with possible but unverified roles in cnidarian symbiosis (see Discussion), we further tested for enrichment of polymorphisms strongly associated with divergence of the Bocas‐specific population in previously identified symbiosis‐related genes. After excluding SNPs in intergenic regions, this analysis was based on 1,432 distinct genes in our dataset that contained one or more high‐quality SNPs with a minor allele frequency of at least 5%. Based on previous studies of symbiosis in *Exaiptasia,* we tested four gene sets for enrichment that had SNP markers in at least three genes in our dataset. These gene sets included a list of scavenger receptors (three genes present; Neubauer, Poole, Weis, & Davy, 2016), genes involved in the complement pathway (three genes present; Baumgarten et al., 2015), genes encoding proteins with thrombospondin‐type‐1 repeat motifs (14 genes present; Neubauer et al., 2017), and genes differentially regulated in larvae during symbiosis establishment (38 genes present; Wolfowicz et al., 2016).

Both thrombospondin‐type‐1 repeat domain‐containing proteins (Neubauer et al., 2017) and genes involved in activating the complement system (Poole, Kitchen, & Weis, 2016) were more likely than expected by chance to exhibit strong associations with PC1 after correcting for multiple comparisons (*p = *.01 and *p *=* *.04, respectively; Table 4). We observed no evidence in our dataset indicating overall enrichment of SNPs strongly associated with PC1 in scavenger receptors (*p *=* *.31; Neubauer et al., 2016) or genes differentially regulated during symbiosis establishment in larvae (*p = *.52; Wolfowicz et al., 2016).

**Table 4:** Genes in symbiosis‐related gene sets enriched for SNP associations with PC1. *F*_ST_ values for each locus are also given. Only loci with *F*_ST_ values in comparisons between the Bocas‐specific and the global *E. pallida* populations above the genomewide median of 0.1 are listed

Classification	Scaffold	Position	Gene ID	Annotation	*F*_ST_
VWA‐domain TSR	46	59254	AIPGENE29239	EGF‐like repeat and discoidin I‐like domain‐containing protein 3	0.88
ADAMTS‐like	105	237954	AIPGENE15743	ADAM‐9	0.77
Complement	90	100854	AIPGENE2377	Complement component 3 (C3)	0.72
VWA‐domain TSR	280	116748	AIPGENE20111	Lactadherin	0.70
ADAMTS‐like	32	161937	AIPGENE3437	ADAMTS‐6	0.66
ADAMTS‐like	207	241923	AIPGENE19810	ADAMTS‐6	0.66
ADAMTS‐like	32	123333	AIPGENE3355	ADAMTS‐6	0.54
ADAMTS‐like	32	79397	AIPGENE3413	ADAMTS‐7	0.51
VWA‐domain TSR	280	122504	AIPGENE20111	Lactadherin	0.38
ADAMTS‐like	78	399268	AIPGENE26625	ADAMTS‐19	0.36
TSR domain only	139	186746	AIPGENE10970	Hemicentin‐1	0.33
Complement	343	122144	AIPGENE1009	Venom factor	0.32
Complement	343	133790	AIPGENE1009	Venom factor	0.14

## 4 DISCUSSION

This study offers a fine‐scale view into ecological and evolutionary dynamics of wild *Exaiptasia* sea anemones. Our findings were consistent with specialization of closely related host species and their symbiont communities for particular habitats. Furthermore, we discovered a distinct, locally abundant population of *E. pallida* that harbored a single symbiont species. Markers present in candidate symbiosis‐related genes were among the most highly differentiated in our dataset, pointing to intraspecific variation in host specificity associated with divergence from the globally distributed population. These findings support an important role of environmental variation in shaping co‐evolutionary dynamics of *Exaiptasia* holobionts at regional scales.

Our results contribute to the broader perspective of genetic diversity emerging for natural *Exaiptasia* populations*,* crucial for interpreting the rapidly growing body of scientific literature surrounding this important laboratory symbiosis system (Goldstein & King, 2016; Hoang, Morran, & Gerardo, 2016). Until recently, evolutionary perspectives on phylogenetic relationships and genetic diversity of the anemone host have been few (but see Thornhill et al., 2013; Bellis et al., 2016; Grajales & Rodríguez, 2016). Focusing on a previously identified geographic area of high diversity, we discovered a unique population of *E. pallida* that may be endemic to the Bocas del Toro Archipelago in Caribbean Panama (Figure 5). This population is genetically distinct from the previously described “global” and “South US Atlantic” networks (Grawunder et al., 2015; Thornhill et al., 2013). In general, the patterns of genetic diversity described here and elsewhere (Bellis et al., 2016; Thornhill et al., 2013) are consistent with a large, genetically diverse population located in the Western Atlantic that has occasionally given rise to new populations throughout the evolutionary history of *Exaiptasia*.

We describe the Bocas‐specific population as genetically distinct; it may represent an incipient species. Some evidence was observed for admixture from the global population (Figure 5a), and despite a large proportion of highly structured polymorphisms (Figure 6), the genomewide median *F*_ST_ between the two populations was moderate (~0.10). There was no indication of variation in mitochondrial sequence, although mitochondrial DNA divergence is not very useful for resolving closely related anthozoan species, because it evolves very slowly (Shearer, Van Oppen, Romano, & Wörheide, 2002). The Bocas‐specific population may have a strong competitive advantage in its home environment, judging by the paucity of individuals from the global population particularly at the inshore site Cayo Roldán (Figure 5a). Anemone population density was higher than any other site sampled (Figures 3), and there was no evidence of reductions in symbiont density when populations were sampled (Figure 4), although Cayo Roldán typically reaches the highest temperatures of the study sites in the Bocas del Toro Archipelago (Figure 2).

We also observed associations between abiotic environment and variation in symbiont community assemblages in natural populations. Clade B1 *Symbiodinium* dominated both *E. pallida* and *E. brasiliensis* individuals throughout the thermally stable Bocas del Toro Archipelago in areas shaded by mangrove canopy and at a shaded location in Galeta (Table 1). In contrast, *E. brasiliensis* harbored at least two genotypes of *Symbiodinium* clade A but not *S. minutum* in a high light, variable temperature site on a shoaling reef flat at Galeta (Table 1). *S. minutum* may therefore be favored in thermally stable, intermediate light environments, with *Symbiodinium* clade A predominating if exposure to high light and temperature is likely. This pattern of distribution is consistent with previously reported photophysiological characteristics of representatives of these *Symbiodinium* lineages (Goulet et al., 2005; Suggett et al., 2015). Similar variation in symbiont type according to abiotic environment is well documented in natural populations of corals (Keshavmurthy et al., 2012; Rowan, Knowlton, Baker, & Jara, 1997).

Abiotic environment and symbiont type were also associated with cryptic host diversity. Both species of *Exaiptasia* were identified in both regions, but *E. pallida* was only found attached to vertical substrate (e.g., mangrove roots or dock pilings), whereas *E. brasiliensis* was more common attached to rocks and coral rubble in Galeta (Table 1). Because no anemones were found on rocks or coral rubble in Bocas del Toro in our study, sampling site was confounded with substrate type. It is therefore unclear to what extent the observed patterns of distribution could result from variation in host preference for different symbiont types, abiotic environments, and/or substrates. However, this study adds to a growing body of research in cnidarians that has uncovered cryptic host diversity associated with both habitat and symbiont type in natural populations of symbiotic cnidarians (Prada et al., 2014; Warner, Van Oppen, & Willis, 2015).

The potential variation in habitat preference described here has important practical implications given the inability to induce larval settlement in the laboratory, a major challenge for further development of *Exaiptasia* as a genetic model system. One possibility is that early uptake of different *Symbiodinium* types could influence the type of substrate upon which anemone larvae prefer to settle. Larval settlement of *Fungia scutaria*, a scleractinian coral that also acquires symbionts horizontally, occurred earlier when larvae were colonized by *Symbiodinium* (Schwarz, Krupp, & Weis, 1999)*. Exaiptasia* larvae similarly take up *Symbiodinium* prior to settlement and metamorphosis (Wolfowicz et al., 2016). Additionally, both temperature and the identity of *ex‐hospite* symbiont types can influence settlement behavior and substrate choice in larvae of broadcast‐spawning corals (Winkler, Pandolfi, & Sampayo, 2015). In the lagoon sites we studied, most propagation beyond individual mangrove roots was attributable to sexual reproduction, implying that *E. pallida* larvae actively seek out shaded, vertical, and/or fibrous substrates for settlement or that they differentially survive in such habitats. Variation in *Exaiptasia* settlement success may also be influenced by the strength of ocean currents at particular sites. Clonal propagation in the Bocas del Toro Archipelago was highest in the exposed offshore site Cayo Agua, suggesting an important role of wave exposure in mediating rates of asexual reproduction and eco‐evolutionary dynamics. Future investigations of variation in habitat preference among *Exaiptasia* populations and species could provide useful guidance for identifying the cues necessary to induce larval settlement under controlled conditions.

Despite a relatively limited genomic sequencing effort, patterns of genetic variation suggested strong differentiation of polymorphisms in putative symbiosis‐related genes. While providing high resolution for population genetic studies, RAD‐based approaches survey only a small proportion of the genome (Lowry et al., 2017). We are therefore likely to have missed the vast majority of loci that may be under selection between the global and Bocas‐specific populations. We expected to sequence at most ~1% of the *E. pallida* reference genome based on *a priori* predicted locations of all *BcgI* tags. Considering blocks of linkage disequilibrium extending approximately 5 kb on either side of genotyped loci as documented for another broadcast‐spawning anthozoan (Shinzato, Mungpakdee, Arakaki, & Satoh, 2015), our callset of 2.6k SNPs might reflect differentiation across up to 10% of the 260 Mb *Exaiptasia* genome. Assuming strong selection and a complex genetic architecture of symbiosis involving hundreds of genes, identification of a handful of symbiosis‐related outlier loci in our study may not be surprising.

Concordantly, outlier loci in previously identified candidate symbiosis genes were among the most highly differentiated SNPs in our dataset. Gene set enrichment analyses revealed overrepresentation of loci in the complement pathway and thrombospondin‐type‐1 repeat (TSR) domain genes (Table 4). A locus in complement C3, a protein that opsonizes foreign microbes and then enhances their phagocytosis by binding to receptors on the surface of host cells (Davy et al., 2012; Poole et al., 2016), was highly differentiated in our dataset. Loci in several TSR domain genes were also highly differentiated. In a previous study, blocking TSR domains decreased colonization of *Exaiptasia* anemones by symbionts, whereas addition of exogenous TSRs resulted in a “supercolonized” phenotype (Neubauer et al., 2017). Most of the differentiated TSR loci in our study were in ADAMTS‐like genes, a family of proteases involved in remodeling of the extracellular matrix (Kelwick, Desanlis, Wheeler, & Edwards, 2015). However, the most differentiated TSR locus was present in EGF‐like repeat and discoidin I‐like domain‐containing protein 3 (a.k.a. DEL‐1), a secreted calcium‐binding molecule that mediates cell adhesion through binding to integrins (Hidai et al., 1998). A role in blocking complement‐dependent phagocytosis in vertebrate immune cells has been proposed for DEL‐1 (Mitroulis et al., 2014).

Our analyses further revealed variation in genes that have not been previously implicated in cnidarian symbiosis. The individual locus most strongly associated with differentiation of the Bocas‐specific population was present in myoferlin (Table 3). Myoferlin is a calcium‐activated protein that regulates lysosomal exocytosis. Myoferlin‐deficient mice accumulate phagolysosomes (Song & Hanayama, 2016), mirroring the early arrest of phagosomal maturation that leads to persistence of symbiosomes within cnidarian cells (Chen, Cheng, Sung, Kuo, & Fang, 2003). Loci in *N*‐glycanase 1 and cytoplasmic FMR1‐interacting protein 2 (CYFIP2) were also strongly associated with divergence of the Bocas‐specific population (Table 3). *N*‐glycanase 1 is an enzyme that cleaves *N‐*linked glycans preferentially from high‐mannose glycoproteins (Zhou et al., 2006) and may be of interest given evidence for an important role of glycan/lectin interactions in recognition of cnidarian symbionts (Davy et al., 2012; Wood‐Charlson, Hollingsworth, Krupp, & Weis, 2006). CYFIP2 is a pro‐apoptotic gene involved in adhesion of human immune cells (Mayne et al., 2004) and is part of WAVE1, a protein complex that activates actin filament reorganization and regulates formation of cytoplasmic projections prior to phagocytosis. These genes may represent interesting targets for further study at cellular levels given their predicted roles in innate immunity, phagocytosis, and regulation of postphagocytic symbiont cell removal.

Although we did not quantify infection rates in this study, differentiation of genes involved in mediating symbiosis could suggest local adaptation of host populations through evolutionary fine‐tuning of interactions with regionally abundant *Symbiodinium*. Theory predicts the existence of co‐evolutionary hotspots where the intensity of reciprocal selection on symbiotic interactions is strong, drift is weak, and patterns of gene flow are conducive to local adaptation (Thompson, 1999). In cnidarian–dinoflagellate symbioses, reciprocal selection is likely to be strong in the tropics, where exogenous nutrients are generally low and irradiance and temperature are high year‐round compared to more temperate latitudes (Muller‐Parker & Davy, 2001). Anemones in the tropics may be more reliant on autotrophy, and *Symbiodinium* densities exhibit greater sensitivity to variation in temperature and irradiance compared to temperate anemone symbioses (Muller‐Parker & Davy, 2001). The extent to which particular regions such as the Bocas del Toro Archipelago may serve as co‐evolutionary hotspots more broadly among symbiotic broadcast‐spawning invertebrates remains to be seen.

In conclusion, this study underscores the importance of characterizing cryptic species diversity in cnidarians and natural variation in laboratory model systems. As global temperatures rise, improved understanding of coral resilience mechanisms such as adaptation or partner shifts is vital for informing management efforts (van Oppen, Oliver, Putnam, & Gates, 2015). Our results emphasize the potential for genetic variation in host specificity not only between cnidarian species but also among populations, and the importance of long‐term evolutionary history in shaping coral–*Symbiodinium* interactions. Integrating studies of natural variation with the extensive molecular resources available for *Exaiptasia* promises to continue providing a wealth of future insights on the cellular, ecological, and evolutionary dynamics of marine symbioses.

## CONFLICT OF INTEREST

None declared.

## AUTHOR CONTRIBUTIONS

ESB contributed to research design, data acquisition, and analysis, and drafted the initial manuscript. RBE and HKB acquired and analyzed data. HAL and DRD contributed to research design, data interpretation, and critical review of the manuscript. All authors read and approved the final manuscript.

## DATA ACCESSIBILITY

DNA sequences are available from GenBank for 2bRAD sequencing reads [NCBI SRA: SRP111971] and Sanger sequences [NCBI GenBank MF942419‐MF942434]. Sampling locations, anemone abundance, *Symbiodinium* cell count, and genotype data files are available through the Dryad Digital Repository (https://doi.org/10.5061/dryad.g8t9240). Custom scripts are available in Appendix S1.
