# Supplementary material for: Molecular signatures of host specificity linked to habitat specialization in Exaiptasia sea anemones
Source: Ecol Evol. 2018 Apr 30;8(11):5413–26. doi: 10.1002/ece3.4058 (PMC6010850; doi:10.1002/ece3.4058)
Supplement: Supplementary file 1 [file ECE3-8-5413-s001.docx]

**Supplemental Information for:**

**Molecular signatures of host specificity linked to habitat specialization in *Exiptasia* sea anemones**

Emily S. Bellis, Reid B. Edlund, Hazel K. Berrios, Harilaos A. Lessios, and Dee R. Denver

**Table of Contents:**

| **Supplementary Materials and Methods** | Page 2 |
| --- | --- |
| **Supplementary Figures: Phylogenetics** | Page 7 |
| **Supplementary Figures: STRUCTURE analyses** | Page 9 |
| **Supplementary Code** | Page 13 |

SUPPLEMENTARY MATERIALS AND METHODS

*Sample storage and processing*

Anemones were collected from Galeta between 15 and 19 July 2015 and from sites in Bocas del Toro between 30 August and 11 September 2015. Anemones were kept alive until processing at Naos Island Laboratories (Panama City, Panama) or the Bocas del Toro Research Station (Colon Island, Panama) that occurred within one week of sample collection. After homogenization in 200 uL Buffer EB (Qiagen, Valencia, CA, USA) with a motorized Pellet Pestle tissue grinder (Kimble Chase, Vineland, NJ, USA) and sterile polypropylene pestle, 100-uL of tissue homogenate was combined with 180 uL Buffer ATL and 20 μL Proteinase K solution (DNeasy Blood and Tissue Kit, Qiagen, Valencia, CA). Samples were digested at 56°C in a water bath according to the manufacturer’s instructions. After digestion, tissue lysates, stable at room temperature for up to 6 months, were transported from Panama to Oregon State University (Corvallis, OR, USA). Extracted DNA was stored at -20°C for ~8 months before genomic library preparation.

Tissue homogenate not aliquoted for DNA extraction was used for determination of algal cell density and mitotic index. Algal cells were pelleted by centrifugation at 5,000 g for 3 minutes. Pellets were resuspended in 500 uL filtered seawater (FSW) and centrifuged once again. The algal pellet and the combined supernatant were frozen for later determination of protein content, algal cell number, and mitotic index.

*Determination of algal cell density and mitotic index*

Cell counts were performed following a protocol available from the Weis Lab at Oregon State University (OSU) (http://people.oregonstate.edu/~weisv/assets/cellcounts.pdf). Briefly, algal pellets were thawed and resuspended in 500 uL filtered seawater (FSW). In most cases, an aliquot of the sample was further diluted with a recorded volume of FSW such that ~25 cells were counted per hemacytometer square. A glass Pasteur pipette was used to load samples into a Bright-Line Improved Neubauer hemacytometer (Hausser Scientific, Horsham, PA, USA), rinsing the pipette three times between samples with FSW and re-mixing each sample before loading the next replicate. Six replicate counts were performed for each sample, and the number of cells, in a counted number of squares, and the number of dividing cells were recorded. Counts were performed on an Olympus BH-2 light microscope at 200x magnification (Galeta samples) or on a Nikon Eclipse E200 light microscope at 100x magnification (Bocas samples). Dividing cells were confirmed at 400x. Cell concentration for each replicate was calculated as follows, where *N* is the total number of cells counted:

$$\frac{N \mathrm{cells}}{squares counted} x \frac{total volume of 2^{\mathrm{nd}} \mathrm{dilution}}{volume of sample aliquot for 2^{\mathrm{nd}} \mathrm{dilution}} x 10={\#\mathrm{cells}}/{\mu L}$$

The cell concentration was multiplied by the total volume of the supernatant to determine the total number of cells in the suspension.

To account for differences in size among anemones, the total number of algal cells was normalized to total protein. Protein content in the supernatant was determined using the Pierce BCA Protein Assay according to the manufacturer’s instructions (Thermo Scientific, Rockford, IL, USA). Absorbance at 562 nm was measured for all samples in duplicate using a Genesys 20 Spectrophotometer (Thermo Scientific, Rockford, IL, USA) at the STRI Bocas del Toro Research Station (Colon Island, Panama). Cell densities were analyzed using a generalized linear model with the Gamma distribution and identity link function in R.

*Preparation of 2bRAD libraries*

Preparation of 2bRAD genomic libraries followed the protocol available from the Meyer lab at OSU (http://people.oregonstate.edu/~meyere/docs/2bRAD_25Aug2016.pdf) and described in detail here. This protocol involves three stages: digestion of DNA with a type IIb restriction enzyme, adaptor ligation, and amplification and barcoding of libraries via PCR. DNA was quantified before digestion using the AccuBlue BroadRange dsDNA Quantitation Kit (Biotium, Inc., Fremont, CA, USA).

To perform DNA digests, 1.2 μg of DNA was concentrated into 8 μL by ethanol precipitation and combined with 4 μL of a digestion master mix comprising 1.2 μL Buffer 3.1, 0.8 μL 150 μM SAM, 0.5 μL BcgI (2 U/μL, New England BioLabs, Ipswich, MA, USA), and 1.5 μL nuclease-free water (NFW). Digestion reactions were incubated for 4 hours at 37°C, and then *BcgI* was heat-inactivated at 65°C for 20 minutes.

To ligate partially double stranded adaptors to 36 bp fragments generated by *BcgI*, 2μM adaptors were prepared by annealing oligonucleotides 5ILL-NN (5’-CTA CAC GAC GCT CTT CCG ATC TNN-3’) and anti-ILL (5’-AGA TCG GAA GAG C[inverted dT]-3’) or 3ILL-NN (5’-CAG ACG TGT GCT CTT CCG ATC TNN-3’) and anti-ILL. Adaptors were annealed for 10 minutes at room temperature before combining 10 μL of digested DNA with 40 μL of the ligation master mix comprising 1 μL 10 mM rATP, 4 μL 10x T4 ligase buffer, 5 μL Adaptor 1, 5 μL Adaptor 2, 1 μL T4 ligase (400 U/μL, New England BioLabs, Ipswich, MA, USA), and 24 μL NFW. Ligations were performed overnight at 16°C.

Individual libraries were then amplified and barcoded during PCR. Dual barcodes were introduced using sample specific oligonucleotides (5’-CAA GCA GAA GAC GGC ATA CGA GAT[BC]GTG ACT GGA GTT CAG ACG TGT GCT CTT CCG AC-3’ and 5’-TGA TAC GGC GAC CAC CGA GAT CTA CAC[BC]ACA CTC TTT CCC TAC ACG ACG CTC TTC CGA TCT-3’), and ligation products were amplified by combining 5 μL of each barcoded oligonucleotide with 40 μL ligation product and 50 μL PCR master mix consisting of 23 μL NFW, 2 μL 10 mM dNTPs, 2 μL each of the universal primers ILL-Lib1 (10 μM, 5’- AAT GAT ACG GCG ACC ACC GA-3’) and ILL-Lib2 (10 μM, 5’-CAA GCA GAA GAC GGC ATA CGA-3’), 20 μL 5x Q5 buffer, and 1 μL Q5 High-Fidelity Polymerase (New England BioLabs, Ipswich, MA, USA). Amplification was performed for 18 cycles using the following profile: 98°C for 5 s, 60°C for 20 s, 72°C for 10 s. PCR products were run on a 2% agarose gel in a 1x sodium borate buffer containing 1 mM EDTA at 220 V for 60 minutes. The target band of 166 bp was cut from the gel and extracted with 40 μL of NFW added to the microcentrifuge tube containing the gel slice. Gel slices were incubated overnight at -80°C and then centrifuged for 20 minutes at 4°C at max 13500 RPM, and the supernatant extracted while pushing the gel slice against the wall of the tube with a pipet tip.

Individual libraries were quantified via qPCR with the SensiFast SYBR Hi-Rox Kit (Bioline, London, UK) and pooled at equimolar quantities. The pooled library was concentrated with the QiaQuick PCR Purification Kit (Qiagen, Valencia, CA, USA) and sequenced as a single-end 37 bp run in high output mode on the NextSeq 500 at the University of Oregon Genomics and Cell Characterization Core Facility (Eugene, OR).

*Genotyping*

Genotype calling was performed for 2bRAD data as previously described (Wang *et al.* 2012) using scripts available from the Meyer lab at OSU except where indicated (https://github.com/Eli-Meyer/2brad_utilities/tree/v2.0). Reads were trimmed from 38 bp to 36 bp to remove adaptor sequence with ‘fastx_trimmer’ (http://hannonlab.cshl.edu/fastx_toolkit). To enable comparisons with previously studied lab strains, we also performed *in silico* digestion of 100 bp paired-end reads previously generated by low-coverage whole genome sequencing of 10 *Aiptasia* strains (Bellis *et al.*, 2016; BcgIDigestFastq.pl script maintained at https://github.com/em-bellis/PanamaPopGen/ and archived here). Reads for which less than 83% of called bases had Phred quality scores less than 28 were removed with ‘fastq_quality_filter’ (http://hannonlab.cshl.edu/fastx_toolkit). Phred quality scores were consistently low at the six invariant bases of the *BcgI* recognition site ([N_10_]CGA[N_6_]TGC[N_12_]); the fastq filtering parameters allowed for low quality at the six invariant bases of the recognition motif, but required high quality at all remaining 30 sites in the read. Low quality at the six invariant sites was associated with a substantial number of miscalled bases at positions 15, 22, and 23 in the read, where median quality was lowest. To improve mapping of reads with miscalled bases at the recognition site, we masked bases at positions 13-15 and 22-24 by bioinformatically changing them to the correct recognition sequence if the correct bases were observed at positions 13 and 14 or at positions 13 and 24. Reads that did not meet these criteria were left unaltered (FixBcgIRecognitionSites.pl maintained at <https://github.com/em-bellis/PanamaPopGen/> and archived here). Before masking, an average of 10.9% of reads from each sample matched the recognition motif exactly. Masking increased the number of reads with exact matches to the recognition motif to 82.7%

After read processing and filtering, reads were aligned to the set of 44,428 *BcgI* recognition sites present in the *Aiptasia* reference genome v1.0 (Baumgarten *et al.* 2015). Mapping was performed using Shrimp v2.2.2, and alignments were filtered to remove ambiguous alignments or weak matches (alignment length < 32 bp or < 30 matching bases). Samples were genotyped as heterozygotes if the minor allele frequency (MAF) at the locus exceeded 30% or as homozygotes if the minor allele frequency at the locus was less than 5%; genotypes with MAF between 5% and 30% or coverage below the thresholds of 5x, 10x, or 15x were not called. Sample BTCA44 was excluded from the final dataset due to low sequencing coverage. Samples CAPG1 and CAPGB4 were also excluded because very few reads from these individuals (<1%) mapped to the reference with high confidence. Sites were excluded if they were not polymorphic in at least 4 individuals (MinPolyFilter.pl from https://github.com/em-bellis/PanamaPopGen/), if minimum allele frequency was less than 5%, if there were more than 2 polymorphisms observed within the same 36 bp tag, or if they were genotyped in less than 80% of individuals.

SUPPLEMENTAL FIGURES

Figure S1. Species assignments based on molecular phylogenetic analysis. Phylogeny was inferred by using the Maximum Likelihood method based on the Kimura 2-parameter model [1]. Bootstrap values (1,000 replicates) are shown next to the corresponding node. The analysis involved 162 samples and was based on a total of 2991 SNPs genotyped in at least 80% of samples with a minimum coverage of 10x. Phylogenetic analyses were conducted in MEGA7 [2]. Not all taxon identifiers are included.

1. Kimura M. (1980). A simple method for estimating evolutionary rate of base substitutions through comparative studies of nucleotide sequences. *Journal of Molecular Evolution* 16:111-120.

2. Kumar S., Stecher G., and Tamura K. (2016). MEGA7: Molecular Evolutionary Genetics Analysis version 7.0 for bigger datasets. *Molecular Biology and Evolution* 33:1870-1874.

Figure S2. Phylogenetic relationships based on DNA sequence spanning a region of cytochrome oxidase III to cytochrome oxidase I. Trees were based on maximum likelihood analysis of 719 bp of mtDNA. Sequenced *E. brasiliensis* included three individuals sampled from the Bocas del Toro Archipelago, one anemone from Galeta, and one anemone from Portobelo. The three sequenced *E. pallida* individuals (BTCA5, BTCA34, BTCR12) were representatives of the Bocas-specific population. Bootstrap values (1,000 replicates) are indicated to the left of the corresponding node. Branch lengths are measured in substitutions per site. Sequences for all identified *E. pallida* individuals were identical to previously reported mitochondrial sequence of *Aiptasia* sp*.* [GenBank accession: HG423148].

Figure S3. Selection of K=2 in the STRUCTURE analysis of the full *E. pallida* dataset.

Figure S4. Selection of K=2 in the STRUCTURE analysis of the Bocas-specific *E. pallida* lineage.

Figure S5. Selection of K=3 in the STRUCTURE analysis of the *E. pallida* global lineage.

Figure S6: STRUCTURE analysis of *E. brasiliensis.* The optimal number of clusters (K=2) was chosen based on the Evanno (2005) method and analyses were performed as described for *E. pallida.*

SUPPLEMENTARY CODE

Additional perl code written by ESB needed to recreate the sample genotyping described here is archived on pages 14-18 of this supplement and maintained at <https://github.com/em-bellis/PanamaPopGen/>.

BcgIDigestFastq.pl

#!/usr/bin/env perl

##Objective: Truncates a set of short reads in FASTQ format to keep only 36 bp regions targeted by BcgI\n"

my $seqfile = $ARGV[0]; # raw reads, fastq format

my $outfile = $ARGV[1]; # name for output file, fastq format

# loop through fastq file and truncate sequences and quality scores

open (IN, $seqfile);

open (OUT, ">$outfile");

my %fastqi;

while(<IN>)

{

chomp;

$count++;

if ($count==1) {$ss = substr($_, 0, 6);}

if ($_ =~ /^$ss/) #is the identifier line

{

$fastqi{id} = $_;

next;

}

if ($_ =~ /^\+$|^\+SRR/)

{

$fastqi{plus} = "+"; #is the + line

next;

}

else

{

$ssi = $_;

if ($ssi =~ /^[ACTGN]+$/) #is the line a sequence line?

{

if ($ssi =~ /(.{12}CGA.{6}TGC.{12}|.{12}GCA.{6}TCG.{12})/)

{

$startpos = $-[0];

$fastqi{seq} = $1;

$printme = "Yes";

}

else #it's a seq line, but doesn't have a BcgI cut site

{

$printme = "No";

}

}

else #it's a quality line

{

if ($printme eq "Yes")

{

$qual = substr($_, $startpos, 36);

print OUT $fastqi{id}."\n".$fastqi{seq}."\n+\n".$qual."\n";

}

}

}

}

close(IN);

close(OUT);

FixBcgIRecognitionSites.pl

#!/usr/bin/env perl

##Objective: This script will 'mask' bases at BcgI recognition site. Bases must match at positions 13 and 14 or 13 and 24 '\n"

my $seqfile = $ARGV[0]; # raw reads, fastq format

my $outfile = $ARGV[1]; # name for output file, fastq format

# loop through fastq file and truncate sequences and quality scores

open (IN, $seqfile);

open (OUT, ">$outfile");

my %fastqi;

while(<IN>)

{

chomp;

$count++;

if ($count==1) {$ss = substr($_, 0, 5);}

if ($_ =~ /^$ss/) #is the identifier line

{

print OUT $_."\n";

next;

}

if ($_ =~ /^\+$/)

{

print OUT $_."\n";

next;

}

else

{

$ssi = $_;

if ($ssi =~ /^[ACTGN]+$/) #is the line a sequence line?

{

if ($ssi =~ /(\w{12})CG\w{1}(\w{6})\w{3}(\w{12})/)

{

print OUT $1.CGA.$2.TGC.$3."\n";

$matchedLines1++;

}

elsif ($ssi =~ /(\w{12})C\w{2}(\w{6})\w{2}C(\w{12})/)

{

print OUT $1.CGA.$2.TGC.$3."\n";

$matchedLines1++;

}

elsif ($ssi =~ /(\w{12})GC\w{1}(\w{6})\w{3}(\w{12})/)

{

print OUT $1.GCA.$2.TCG.$3."\n";

$matchedLines2++;

}

elsif ($ssi =~ /(\w{12})G\w{2}(\w{6})\w{2}G(\w{12})/)

{

print OUT $1.GCA.$2.TCG.$3."\n";

$matchedLines2++;

}

else

{

$unmatchedLines++;

print OUT $_."\n";

}

}

else #it's a quality line

{

print OUT $_."\n";

}

}

}

print $matchedLines1." sequences matched recognition site CGANNNNNNTGC.\n";

print $matchedLines2." sequences matched recognition site GCTNNNNNNACG.\n";

print $unmatchedLines." sequences did not match either recognition site at positions 13 and 14 or 13 and 24.\n";

close(IN);

close(OUT);

MinPolyFilter.pl

#!/usr/bin/perl

##read in a table of SNPs called from Meyer lab's 2bRAD pipeline

##filter out SNPs that are not polymorphic in a minimum # of samples. Heterozygotes are also considered polymorphic (i.e. if three individuals are A/A A/G and G/G, the SNP will pass if $ARGV[1] is 2.)

# -- program description and required arguments

unless ($#ARGV == 2)

{print "\nReads in a table of SNPs called from e. meyer's 2bRAD pipeline and filters out SNPs that are not polymorphic in a minimum # of samples\n";

print "Usage:\t MinPolyFilter.pl in.tab 5 out.vcf\n";

print "Arguments:\n";

print "\t table\t\t file of SNPs to be filtered, samples in columns, snps in rows\n";

print "\t minimum number of samples\t SNPs present in fewer individuals than this will be excluded\n";

print "\t output\t\t a name for the output file (vcf format)\n";

print "\n"; exit;

}

#-- take in arguments passed from command line, open files for reading and writing

$infile = $ARGV[0];

$MinIndividuals = $ARGV[1];

$outfile = $ARGV[2];

print("Input file is ", $infile, "\n");

print("Output file is ", $outfile, "\n");

open(INFILE, "<", $infile) || die ("Can't find the file $infile: $!");

open(OUTFILE, ">", $outfile);

#print header lines to the outfile

$header = <INFILE>;

chomp($header);

print OUTFILE $header."\n";

$catsnps = "";

while ($line = <INFILE>) {

chomp($line);

$totalLines++;

@lineParts = split(/\t/, $line);

foreach $i(2..$#lineParts) {

if ($lineParts[$i]=~/[AGCT]/) {

$catsnps = $catsnps.$lineParts[$i];

}

}

my @Acount = ($catsnps =~ /A/g);

my @Gcount = ($catsnps =~ /G/g);

my @Ccount = ($catsnps =~ /C/g);

my @Tcount = ($catsnps =~ /T/g);

my @counts = (scalar @Acount, scalar @Gcount, scalar @Ccount, scalar @Tcount);

foreach $j(0..$#counts) {

if ($counts[$j] >= $MinIndividuals) {

$passes++;

}

}

if ($passes >1){

print OUTFILE $line."\n";

$passingLines++;

}

$passes=0;

$catsnps="";

}

print $passingLines." SNPs were present in at least ".$MinIndividuals." individuals.\n";

print $totalLines." SNPs total were in the file.\n";

close(INFILE);

close(OUTFILE);

countHetSites.pl

#!/usr/bin/perl

##Objective: based on a vcf file, count all the genotyped and heterozygous sites for an individual

my $vcfFile = $ARGV[0];

my $indNumber = $ARGV[1];

open (IN, $vcfFile);

$homSites=0;

$hetSites=0;

while($line = <IN>) {

chomp($line);

if ($line =~ /^#CHROM/) {

@lineParts = split(/\t/,$line);

$indName = $lineParts[$indNumber];

} elsif ($line =~ /^scaffold/) {

@lineParts = split(/\t/,$line);

$genotype = $lineParts[$indNumber];

if ($genotype =~ /0\/0|1\/1|2\/2/) { ##NOTE: output from E. Meyer's gt2vcf.pl script has 1's and 2's instead of 0's and 1's if reference is heterozygous

$homSites++;

} elsif ($genotype =~ /0\/1|1\/0|0\/2|2\/0/) {

$hetSites++;

}

}

}

print $indName."\t".$hetSites."\t".$homSites."\n";

close(IN);
